# GluK2 Q/R editing regulates kainate receptor signaling and long-term potentiation of AMPA receptors

**DOI:** 10.1016/j.isci.2023.107708

**Published:** 2023-08-25

**Authors:** Jithin D. Nair, Kevin A. Wilkinson, Busra P. Yucel, Christophe Mulle, Bryce Vissel, Jack Mellor, Jeremy M. Henley

**Affiliations:** 1 Centre for Synaptic Plasticity, School of Biochemistry, Centre for Synaptic Plasticity, Biomedical Sciences Building, University of Bristol, University Walk, Bristol BS8 1TD, UK; 2 Centre for Synaptic Plasticity, School of Physiology, Pharmacology and Neuroscience, Biomedical Sciences Building, University of Bristol, University Walk, Bristol BS8 1TD, UK; 3 CNRS UMR 5297, Interdisciplinary Institute of Neuroscience, University of Bordeaux, France; 4 Centre for Neuroscience and Regenerative Medicine, St Vincent’s Hospital, Sydney, NSW, Australia

**Keywords:** Biological sciences, Neuroscience, Cellular neuroscience

## Abstract

Q/R editing of the kainate receptor (KAR) subunit GluK2 radically alters recombinant KAR properties, but the effects on endogenous KARs *in vivo* remain largely unexplored. Here, we compared GluK2 editing-deficient mice that express ∼95% unedited GluK2(Q) to wild-type counterparts that express ∼85% edited GluK2(R). At mossy fiber-CA3 (MF-CA3) synapses GluK2(Q) mice displayed increased postsynaptic KAR function and KAR-mediated presynaptic facilitation, demonstrating enhanced ionotropic function. Conversely, GluK2(Q) mice exhibited reduced metabotropic KAR function, assessed by KAR-mediated inhibition of slow after-hyperpolarization currents (I_SAHP_). GluK2(Q) mice also had fewer GluA1-and GluA3-containing AMPA receptors (AMPARs) and reduced postsynaptic AMPAR currents at both MF-CA3 and CA1-Schaffer collateral synapses. Moreover, long-term potentiation of AMPAR-mediated transmission at CA1-Schaffer collateral synapses was reduced in GluK2(Q) mice. These findings suggest that GluK2 Q/R editing influences ionotropic/metabotropic balance of KAR signaling to regulate synaptic expression of AMPARs and plasticity.

## Highlights

• GluK2(Q) mice display enhanced EPSC_KA_ and reduced EPSC_AMPA_
• Mutant mice show a global reduction in GluA1 and GluA3 subunits of AMPARs
• KAR mediated inhibition of I_sAHP_ is reduced in GluK2(Q) mice
• LTP at Schaffer collateral-CA1 synapses is reduced in mutant mice

## Graphical abstract

**Figure:**
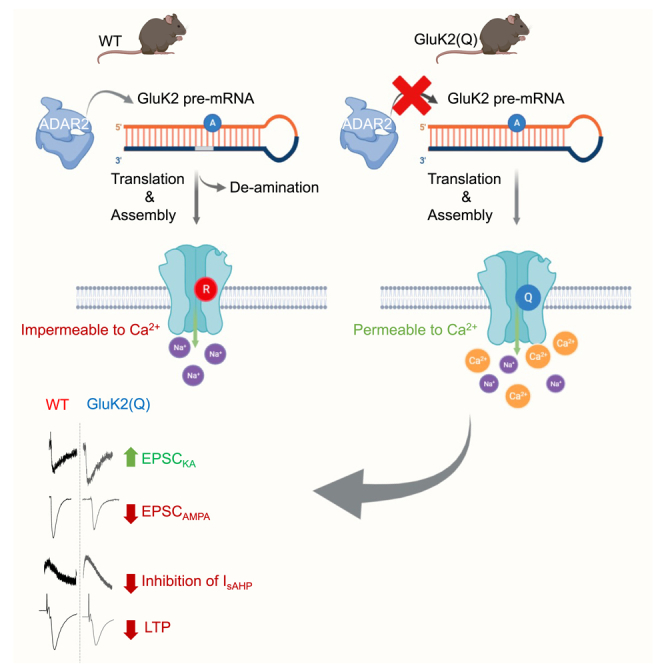

## Introduction

Kainate receptors (KARs) are glutamate-gated cation channels assembled from tetrameric combinations of the subunits GluK1-GluK5. Depending on the synapse and neuron type, KARs are present at both pre- and postsynaptic sites throughout the brain.^1^ Despite their close homology to AMPA- and NMDA-type glutamate receptors, postsynaptic KARs mediate only a minor fraction of the ionotropic synaptic response to glutamate, but they are critically important for synaptic integration and regulation of neural circuits.^2^^,^^3^^,^^4^ Presynaptic KARs also contribute to neuronal network function by regulating neurotransmitter release probability at both excitatory and inhibitory synapses.^5^^,^^6^^,^^7^^,^^8^^,^^9^^,^^10^^,^^11^^,^^12^

KARs have been particularly well studied at hippocampal glutamatergic MF-CA3 synapses,^13^^,^^14^ where the GluK2 subunit is an integral component of both pre- and postsynaptic KARs.^6^^,^^7^^,^^8^^,^^15^^,^^16^^,^^17^ Indeed, ionotropic postsynaptic KARs containing GluK2 were first discovered at MF-CA3 synapses,^13^^,^^14^^,^^15^ and GluK2-containing KARs were shown to contribute to short-term plasticity of presynaptic release probability over timescales ranging from 10ms to 20s.^18^^,^^19^

In addition to ionotropic actions, KARs also initiate G protein-coupled metabotropic signaling.^20^^,^^21^^,^^22^^,^^23^^,^^24^ Metabotropic signaling through postsynaptic KARs has been demonstrated at Schaffer collateral-CA1 synapses^25^ and at MF-CA3 synapses.^16^^,^^26^ Synaptic activation of postsynaptic KARs inhibits the slow after hyperpolarization (I_sAHP_), a long-lasting voltage-independent and Ca^2+^-dependent K^+^ current produced following short bursts of action potentials.^27^ KAR-mediated inhibition of I_sAHP_ occurs in multiple neuronal types via a G_i/o_ G protein and PKC-dependent pathway and, in CA3 cells, is absent in GluK2 knockout mice, suggesting a crucial role for this subunit in this form of metabotropic signaling.^16^^,^^25^^,^^26^^,^^28^ Furthermore, pharmacological activation of KARs by exogenous agonists regulates presynaptic release of both GABA and glutamate through this metabotropic pathway.^20^^,^^29^^,^^30^^,^^31^^,^^32^

KAR surface expression is activity-dependently and bidirectionally regulated.^24^^,^^33^^,^^34^^,^^35^^,^^36^^,^^37^ Furthermore, activation of KARs can also up- or down-regulate AMPAR surface expression to mediate plasticity.^24^^,^^38^ Specifically, transient activation of GluK2-containing KARs in cultured hippocampal neurons increases AMPAR surface expression, and in hippocampal slices GluK2-containing KARs can induce AMPAR long-term potentiation (KAR-LTP_AMPAR_) at Schaffer collateral-CA1 synapses via a pertussis toxin-sensitive metabotropic signaling pathway.^24^ In contrast, sustained activation of KARs reduces surface expression of AMPARs in cultured hippocampal neurons and induces AMPAR long-term depression in CA1 neurons in hippocampal slices (KAR-LTD_AMPAR_), an effect that is lost in the absence of GluK2.^38^ These results highlight the importance of GluK2-containing KARs as modulators of AMPAR-mediated synaptic transmission.

The nuclear enzyme ADAR2 Q/R edits GluK2 pre-mRNA, resulting in a genetically encoded glutamine (Q) in the channel pore region being replaced by an arginine (R).^39^^,^^40^ In recombinant systems KARs containing GluK2(R) subunits display ER retention and reduced traffic to the cell surface compared to those assembled with the unedited GluK2(Q).^41^^,^^42^ Furthermore, the edited GluK2(R)-containing KARs that do reach the surface do not gate Ca^2+^ and have a channel conductance <1% of GluK2(Q).^43^

The dynamic regulation of GluK2 Q/R editing underpins KAR homeostatic plasticity whereby chronic suppression of network activity decreases ADAR2 levels. This, in turn, reduces GluK2 editing resulting in enhanced KAR surface expression. Reciprocally, chronic enhancement of network activity promotes GluK2 Q/R editing and reduces KAR surface expression.^35^^,^^36^^,^^37^

**Figure 1:**
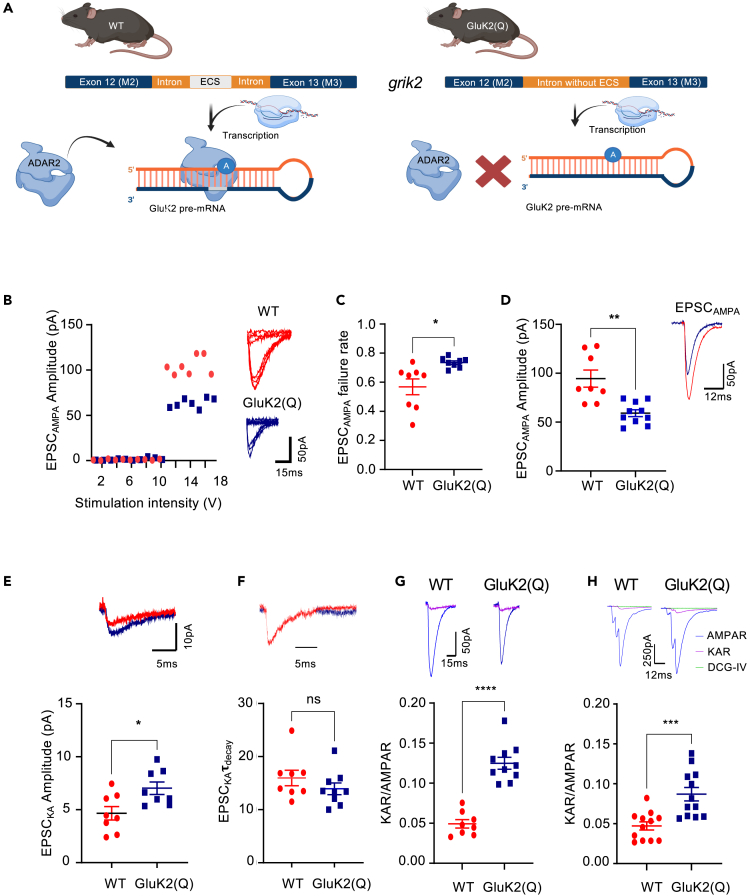
Enhanced postsynaptic KAR and reduced AMPAR currents at MF-CA3 synapses in GluK2(Q) mice (A) The intronic region between exon 12 (M2) and 13 (M3) in the *grik2* gene contains an editing complementary site (ECS), located ∼1900nt downstream of exon 12. In the WT mice, this region is intact. However, in homozygous GluK2(Q) mice, a 600bp region is deleted from the ECS site. This prevents ADAR2 binding and subsequent editing of GluK2 pre-mRNA at the Q/R site, leading to the translation of >95% unedited GluK2(Q) subunits. (B) Representative data from a minimal stimulation experiment showing stimulation intensity threshold for evoking responses from excitation of a single axon fiber (left). Eight superimposed consecutive traces showing synaptic successes and failures for minimal stimulation for WT (red) (top right) and GluK2(Q) mice (blue) (bottom right). (C) Quantification of probability of failures for AMPAR responses out of 150 stimulations in WT and GluK2(Q) mice. N = 5, n = 8 cells for WT and N = 5, n = 10 cells for GluK2(Q) mice; ns p > 0.05, ∗p < 0.05, ∗∗p < 0.01; unpaired t-test with Welch’s correction. (D) Quantification of average amplitude of EPSC_AMPA_ (left). Representative trace showing EPSC_AMPA_ in WT (red) and GluK2(Q) mice (blue) (right).N = 5, n = 8 cells for WT and N = 5, n = 10 cells for GluK2(Q) mice (bottom).; ns p > 0.05, ∗p < 0.05, ∗∗p < 0.01; unpaired t-test with Welch’s correction. (E) Representative trace showing EPSC_KA_ in WT (red) and GluK2(Q) mice (blue) (top). Quantification of average amplitude of EPSC_KA_ (bottom). N = 5, n = 8 cells for WT and N = 5, n = 10 cells for GluK2(Q) mice (bottom); ns p > 0.05, ∗p < 0.05, ∗∗p < 0.01; unpaired t-test with Welch’s correction. (F) Representative trace showing **τ**_decay_ for KAR currents (WT scaled to Tg) in WT (red) versus GluK2(Q) mice (blue) (top) Quantification of **τ**_decay_ for KAR currents in WT vs. GluK2(Q) mice. N = 5, n = 8 cells for WT and N = 5, n = 10 cells for GluK2(Q) mice (bottom); ns p > 0.05, ∗p < 0.05, ∗∗p < 0.01; unpaired t-test with Welch’s correction. (G) Representative traces showing postsynaptic AMPAR (blue) and KAR (purple) currents in WT and GluK2(Q) mice with minimal stimulation (top). Quantification of KAR/AMPAR current ratio in WT (red) and GluK2(Q) mice (blue) (bottom) N = 5, n = 8 cells for WT and N = 5, n = 10 cells for GluK2(Q) mice, ns p > 0.05, ∗∗∗∗p < 0.0001; unpaired t-test with Welch’s correction. (H) Representative traces showing postsynaptic AMPAR (blue) and KAR (purple) currents in WT and GluK2(Q) mice with burst stimulation at 167Hz (top). Quantification of KAR/AMPAR current ratio in WT (red) and GluK2(Q) mice (blue) (bottom). N = 5, n = 12 cells, ns p > 0.05, ∗p < 0.05, ∗∗p < 0.01; unpaired t-test with Welch’s correction.

We used electrophysiology and biochemistry approaches to investigate how GluK2 Q/R editing alters KAR signaling and function in intact neuronal circuits and whether these changes, in turn, regulate AMPAR function. GluK2 editing-deficient mice contain a deletion in the intronic editing complementary sequence (ECS) of the *grik2* gene that directs ADAR2-mediated codon substitution in the GluK2 pre-mRNA (Figure 1A). This results in >95% of GluK2-KARs in adult mice containing unedited GluK2(Q), whereas KARs in WT mice contain <15% GluK2(Q).^44^ Importantly, however, Q/R editing of the GluK1 KAR subunit, and the GluA2 AMPAR subunit, are un-altered in these mice.^44^ GluK2(Q) mice are viable and, as expected, surface expressed KARs in cultures from these mice display the inwardly rectifying current/voltage (I-V) relationship characteristic of GluK2(Q)-containing KARs expressed in recombinant systems.^45^^,^^46^ The only previous study using these GluK2(Q) mice detected no differences in GluK2 mRNA levels, no alterations in editing of the other GluK2 editing sites (I/V and Y/C) and no change in Q/R editing of the GluA2 AMPAR subunit.^44^ However, the GluK2(Q) mice were reported to have increased susceptibility to kainate-induced seizures and display a form of NMDAR-independent LTP at the medial perforant-DG synapses that is not present in WT mice.^44^

Here, we show enhanced pre- and postsynaptic KAR ionotropic function in the GluK2(Q) mice compared to WT controls whereas metabotropic KAR-mediated inhibition of I_sAHP_ was reduced. Furthermore, in GluK2(Q) mice AMPAR-mediated transmission at hippocampal CA3 and CA1 synapses, synaptic levels of GluA1 and GluA3 were decreased and LTP at CA1 Schaffer collateral synapses was severely attenuated. We interpret these data to indicate that GluK2 editing is important for determining the mode of KAR signaling, and that this, in turn, plays key roles in the regulation of basal expression of synaptic AMPARs and AMPAR-mediated synaptic plasticity.

## Results

### Enhanced postsynaptic KAR and reduced AMPAR currents at MF-CA3 synapses in GluK2(Q) mice

In recombinant systems KARs containing unedited GluK2(Q) have a higher conductance than edited GluK2(R).^43^^,^^47^ Moreover, disrupting ADAR2-mediated GluK2 Q/R editing enhances the surface expression, single channel conductance, and Ca^2+^ permeability of postsynaptic KARs in cultured neurons.^36^^,^^43^^,^^48^ Therefore, we measured basal KAR-mediated postsynaptic responses at MF-CA3 synapses in WT and GluK2(Q) mice. Because both the glutamate receptor complement and presynaptic facilitation at MF-CA3 synapses are stable after the 2^nd^ postnatal week^49^^,^^50^ we used P14-P21 mice and confirmed the stability of AMPAR and KAR expression within this age range.

To compare between GluK2-editing deficient and WT genotypes, we used a well-established minimal stimulation protocol to isolate monosynaptic responses at 3Hz in acutely prepared hippocampal slices.^49^^,^^51^^,^^52^^,^^53^ Postsynaptic responses were recorded from CA3 pyramidal neurons in whole-cell voltage clamp configuration in the presence of picrotoxin (50μM) and D-APV (50μM) to block GABA_A_Rs and NMDARs, respectively. The stimulation intensity applied to presynaptic axons was increased in small increments until a postsynaptic response was observed (Figure 1B). Interestingly, the percentage of trials that evoked a synaptic response (success rate) was decreased in the GluK2(Q) mice (Figure 1C) (WT = 43 ± 5%, GluK2(Q) = 27 ± 1%; unpaired t-test, p = 0.017), suggesting either a decrease in the number of release sites or reduced probability of glutamate release from the same number of sites. Furthermore, the average AMPAR-EPSC amplitude for successful trials was reduced in GluK2(Q) mice (Figure 1D) (WT = 94.5 ± 8.8pA, GluK2(Q) = 59.1 ± 3.5pA; unpaired t-test, p = 0.0044).

The AMPAR antagonist GYKI53655 (40μM) was then applied to pharmacologically isolate KAR-mediated excitatory postsynaptic currents (EPSC_KA_) evoked by single presynaptic mossy fiber axons. Analysis of detectable synaptic responses revealed that EPSC_KA_ was increased in amplitude in GluK2(Q) mice (Figure 1E) (WT = 4.66 ± 0.63pA, GluK2(Q) = 7.31 ± 0.50pA; unpaired t-test, p = 0.0057), but with no change in decay kinetics (Figure 1F) (**τ**_decay,_ WT = 16.0 ± 1.5ms, GluK2(Q) = 13.9 ± 1.1ms; unpaired t-test, p = 0.28), consistent with enhanced conductance of unedited GluK2(Q)-containing recombinant KARs.^43^^,^^47^

We also measured the EPSC_KA_ to EPSC_AMPA_ amplitude ratio for both minimal and larger EPSCs evoked by bursts of 3 stimuli at 167Hz given to mossy fiber axons that stimulate multiple axons.^54^ This stimulation protocol was chosen to maximize the KAR response.^26^ EPSC_AMPA_ was first collected in the presence of picrotoxin (50μM) and D-APV (50μM) to block GABA_A_Rs and NMDARs, respectively. Then, EPSC_AMPA_ was blocked by bath application of GYKI53655 (40μM) for 10 min to isolate EPSC_KA_. The EPSC_KA_/EPSC_AMPA_ ratio increased in GluK2(Q) mice (Figures 1G and 1H) (minimal stimulation: WT = 0.049 ± 0.005, GluK2(Q) = 0.12 ± 0.007; unpaired t-test, p = 0.0001; 167Hz stimulation: WT = 0.047 ± 0.005, GluK2(Q) = 0.086 ± 0.008; unpaired t-test, p = 0.0008). Based on the minimal stimulation data for EPSC_KA_ and EPSC_AMPA_, this increase in KAR/AMPAR ratio likely results from an increase in EPSC_KA_, and a decrease in EPSC_AMPA_.

### Enhanced presynaptic facilitation in GluK2(Q) mice

**Figure 2:**
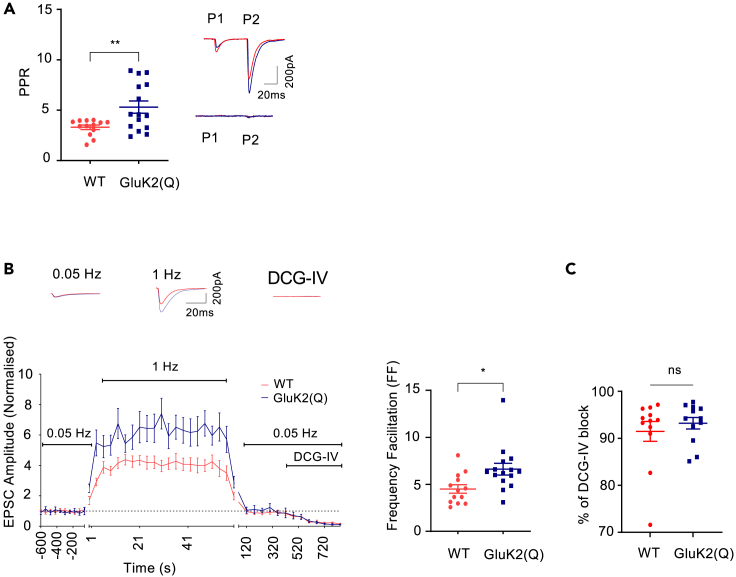
Enhanced presynaptic facilitation in GluK2(Q) mice (A) Quantification of average paired-pulse ratio in WT (red) and GluK2(Q) mice (blue)(left). Representative traces showing EPSCs in response to paired-pulse stimulation from both WT and GluK2(Q) mice before (top right) and after DCG-IV application (bottom right). WT, N = 7, n = 13 cells; GluK2(Q) mice, N = 8, n = 15 cells; ns p > 0.05, ∗p < 0.05, ∗∗p < 0.01; unpaired t-test with Welch’s correction. (B) Representative trace showing frequency facilitation from WT and GluK2(Q) mice (top). Timeline of frequency facilitation experiments in WT and GluK2(Q) mice (bottom left). Quantification of frequency facilitation in WT and GluK2(Q) mice (bottom right). (C) Percentage of DCG-IV block in WT and GluK2(Q) mice. WT, N = 7, n = 13 cells; GluK2(Q) mice, N = 8, n = 15 cells; ns p > 0.05, ∗p < 0.05, ∗∗p < 0.01; unpaired t-test with Welch’s correction.

Presynaptic KARs at MF-CA3 synapses are autoreceptors activated by released glutamate to facilitate the probability of vesicle release in response to subsequent action potentials.^8^^,^^55^^,^^56^^,^^57^ To investigate how GluK2 Q/R editing affects presynaptic KAR function at MF-CA3 synapses, we measured short-term facilitation of presynaptic release in the presence of picrotoxin (50μM) across a range of timescales. By measuring EPSC_AMPA_, we examined paired-pulse facilitation (PPF), observed at 50ms stimulation intervals, and accumulation of frequency facilitation (FF), observed by changing the frequency of stimulation from 50ms to 1s intervals, in acute slices from WT and GluK2(Q) mice using previously described protocols.^15^^,^^18^^,^^19^^,^^58^ Both PPF and FF were increased in GluK2(Q) mice (Figures 2A and 2B) (PPF: WT = 3.3 ± 0.2, GluK2(Q) = 5.3 ± 0.6; un-paired t-test, p = 0.0067; FF: WT = 4.5 ± 0.4, GluK2(Q) = 6.6 ± 0.6; unpaired t-test, p = 0.0114), consistent with enhanced presynaptic KAR function and/or decreased basal release probability.

In all experiments the purity of mossy fiber input was determined by addition of the group II metabotropic glutamate receptor agonist DCG-IV (2μM)^59^ and recordings were excluded if there was <70% inhibition of synaptic responses. Furthermore, to exclude the possibility that differences in the purity of mossy fiber inputs contributed to the enhanced short-term facilitation in GluK2(Q) mice we analyzed the degree of DCG-IV inhibition. No differences were observed (Figure 2C) (WT = 91.5 ± 2.1%, GluK2(Q) = 93.2 ± 1.2%; unpaired t-test, p = 0.4879) and there was no correlation between degree of inhibition by DCG-IV and PPR (Figures S1A and S1B) (WT, r = 0.277, R^2^ = 0.0769, p = 0.383; GluK2(Q), r = −0.280, R^2^ = 0.0784, p = 0.378; 95% confidence interval). There was also no correlation between EPSC amplitude and PPR (Figures S1C and S1D) (WT, r = 0.127, R^2^ = 0.0163, p = 0.692; GluK2(Q), r = 0.0383, R^2^ = 0.00147, p = 0.906; 95% confidence interval). EPSC initial amplitudes in response to the first stimulus were set to be similar between genotypes (WT = 156.6 ± 14.4pA, GluK2(Q) = 143.9 ± 17.8pA; unpaired t-test, p = 0.5830).

Taken together with the reduced success rate observed in the minimal stimulation experiments (Figure 1C), these data suggest that basal release probability is reduced, and presynaptic facilitation is enhanced, at MF-CA3 synapses in GluK2(Q) mice.

### Metabotropic KAR function is impaired in GluK2(Q) mice

**Figure 3:**
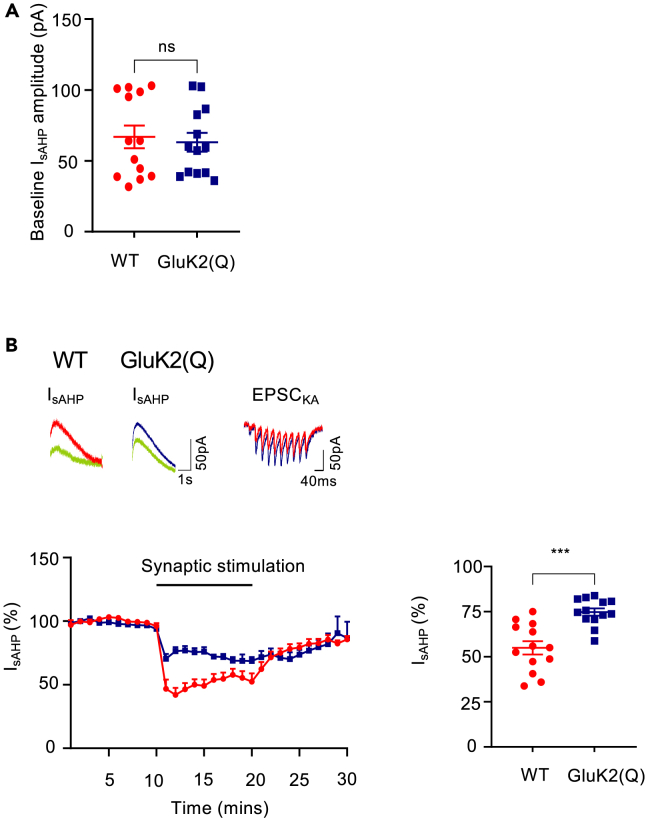
Impaired metabotropic KAR function in GluK2(Q) mice (A) Average baseline amplitude of I_sAHP_ currents in WT (red) and GluK2(Q) (blue) mice. (B) Sample traces from WT before (red) and after synaptic KAR stimulation (green) (top left) and GluK2(Q) mice (blue) before and after synaptic KAR stimulation (top middle) and EPSC_KA_ following synaptic stimulation (top right). Timeline showing inhibition of I_sAHP_ following synaptic KAR activation (bottom left). Quantification of percentage inhibition of I_sAHP_ following synaptic KAR activation in WT and GluK2(Q) mice (bottom right). N = 4 animals, n = 13 cells, ns p > 0.05, ∗∗p < 0.0001; un-paired t-test with Welch’s correction.

Because the GluK2 Q/R editing site is within the channel pore region it is unsurprising that editing impacts on KAR ionotropic signaling but the effects of Q/R editing on metabotropic signaling have not been investigated. We therefore assessed if GluK2 Q/R editing alters metabotropic function by measuring KAR inhibition of the slow afterhyperpolarization current (I_sAHP_) in acute hippocampal slices.^16^^,^^25^^,^^26^^,^^60^ I_sAHP_ currents were evoked in whole-cell voltage clamped CA3 pyramidal cells by depolarizing the membrane potential to 0mV from −50mV for 200ms in the presence of 50μM picrotoxin, 50μM D-APV, 40μM GYKI53655 and 1μM CGP55845 to inhibit GABA_A_Rs, NMDARs, AMPARs and GABA_B_Rs, respectively.^26^ Robust and stable I_sAHP_ currents were obtained in both WT and GluK2(Q) mice, with no difference in baseline amplitudes between genotypes (Figure 3A) (WT = 67.0 ± 8.0pA, GluK2(Q) = 63.1 ± 6.6pA; unpaired t-test, p = 0.712). Activation of synaptic KARs by MF stimulation (10 stimuli at 25Hz every 20 s for 10 mins), as previously described,^26^^,^^61^ produced a consistent depression of I_sAHP_ in WT mice but the extent of depression was decreased in GluK2(Q) mice (Figure 3B) (WT = 45.1 ± 3.7%, GluK2(Q) = 26.7 ± 2.7%; unpaired t-test, p = 0006). These data indicate that KAR metabotropic signaling is impaired in GluK2(Q) mice.

### Synaptic expression of KAR subunits is altered in GluK2(Q) mice

**Figure 4:**
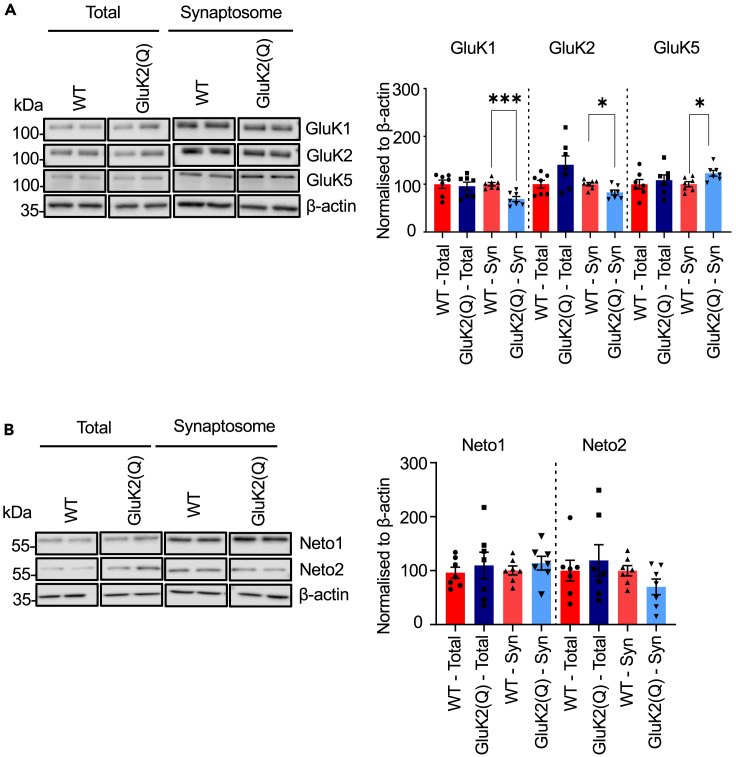
Altered synaptic KAR subunit expression in GluK2(Q) mice (A) Representative Western blots of total and synaptosomal fraction samples from a single cerebral hemisphere of WT or GluK2(Q) mice for KAR subunits (left). Quantification of proteins expressed as percentage of WT protein after normalizing to β -actin (right). β-actin was used as a loading control. N = 7 animals; ns p > 0.05, ∗p < 0.05, ∗∗p < 0.01; unpaired t-test with Welch’s correction. (B) Representative Western blots of total and synaptosomal fraction samples from a single cerebral hemisphere of WT or GluK2(Q) mice for auxiliary KAR subunits Neto1 and Neto2 (left) Quantification of proteins expressed as percentage of WT protein after normalizing to β-actin (right). β-actin was used as a loading control N = 7; ns p > 0.05, ∗p < 0.05, ∗∗p < 0.01; unpaired t-test with Welch’s correction.

The reduced metabotropic signaling in GluK2(Q) mice could arise from disrupted molecular signaling and/or reduced expression of synaptic KARs. We therefore assessed total and synaptosomal expression of key KAR subunits in WT and GluK2(Q) mice by western blotting. Total expression of the GluK1, GluK2 and GluK5 was unaltered in GluK2(Q) mice (Figure 4A) (GluK1: WT = 100 ± 8.6%, GluK2(Q) = 95.7. ± 8.5%, unpaired t-test, p = 0.73; GluK2: WT = 100 ± 8.1%, GluK2(Q) = 140.6 ± 18.5 unpaired t-test, p = 0.07; GluK5: WT = 100.0 ± 9.8%, GluK2(Q) = 108.4 ± 10.8%, unpaired t-test, p = 0.57).

In synaptosomes, however, the expression of GluK1 and GluK2 was reduced whereas expression of GluK5 was increased (Figure 4A) (GluK1: WT = 100 ± 351%, GluK2(Q) = 69.0 ± 5.3%, unpaired t-test, p = 0.0006; GluK2: WT = 100 ± 3.5%, GluK2(Q) = 82.7 ± 5.1, unpaired t-test, p = 0.002; GluK5: WT = 100.0 ± 5.3%, GluK2(Q) = 122 ± 6.0%, unpaired t-test, p = 0.01). These data indicate that the enhanced pre- and postsynaptic ionotropic KAR function demonstrated in (Figures 1 and 2) are likely attributable to the increased conductance of GluK2(Q)-containing KARs, rather than an increase in the number of synaptic KARs.

Since most postsynaptic KARs comprise heteromeric combinations of GluK2/GluK5 and the auxiliary subunits Neto1 and/or Neto2, depending on the cell type,^62^ we next investigated the expression levels of the Neto1 and Neto2. Neither total (Neto1: WT = 100 ± 10.5%, GluK2(Q) = 114.0 ± 25.4%, unpaired t-test, p = 0.62; Neto2: WT = 100 ± 19.1%, GluK2(Q) = 118.9 ± 29.0%, unpaired t-test, p = 0.59) nor synaptic expression of Neto1 and Neto2 were altered in the GluK2(Q) mice (Neto1: WT = 100 ± 8.17%, GluK2(Q) = 113.4 ± 12.6%, unpaired t-test, p = 0.39; Neto2: WT = 100 ± 9.4%, GluK2(Q) = 69.8 ± 14.4%, unpaired t-test, p = 0.11) (Figure 4B),.

### Reduced synaptic AMPAR expression in GluK2(Q) mice

**Figure 5:**
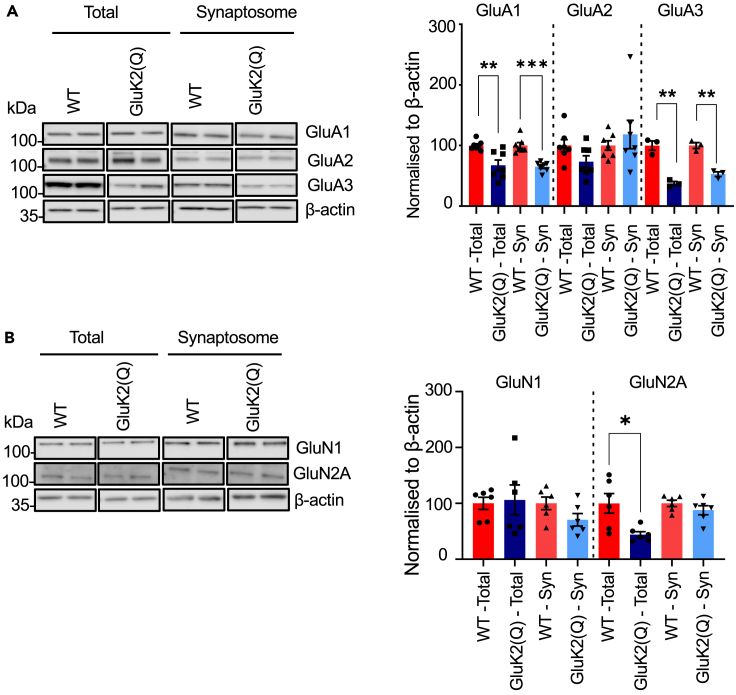
Reduced synaptic AMPAR expression in GluK2(Q) mice (A) Representative Western blots of total and synaptosomal fraction samples from a single cerebral hemisphere of WT or GluK2(Q) mice for the AMPAR subunits GluA1-3 (left). Quantification of proteins expressed as percentage of WT protein after normalizing to β-actin (right). β-actin was used as a loading control. N = 7 animals; ns p > 0.05, ∗p < 0.05, ∗∗p < 0.01; unpaired t-test with Welch’s correction. (B) Representative Western blots of total and synaptosomal fraction samples from a single cerebral hemisphere of WT or GluK2(Q) mice for the NMDAR subunits GluN1 and GluN2A (left). Quantification of proteins expressed as percentage of WT protein after normalizing to β-actin (right). β-actin was used as a loading control N = 6 animals (B); ns p > 0.05, ∗p < 0.05, ∗∗p < 0.01; unpaired t-test with Welch’s correction.

Given that GluK2(Q) mice display a reduction in the EPSC_AMPA_ (Figure 1D) we next stripped the same blots and reprobed for total and synaptic expression of AMPARs. Intriguingly, total (GluA1: WT = 100 ± 2.9%, GluK2(Q) = 67.4 ± 8.7%, unpaired t-test, p = 0.009; GluA3: WT = 100 ± 6.5%, GluK2(Q) = 37.3 ± 2.8%, unpaired t-test, p = 0.006) and synaptosomal levels of the AMPAR subunits GluA1 and GluA3 were reduced, (GluA1: WT = 100 ± 4.8%, GluK2(Q) = 65.1 ± 3.0%, unpaired t-test, p = 0.0001; GluA3: WT = 100 ± 4.2%, GluK2(Q) = 52.7 ± 3.3%, unpaired t-test, p = 0.002) (Figure 5A). In contrast, levels of GluA2 were unchanged in both total (WT = 100 ± 9.9%, GluK2(Q) = 73.4 ± 9.6%, unpaired t-test, p = 0.08) and synaptosome fractions (WT = 100 ± 7.7%, GluK2(Q) = 118.2 ± 23.4%, unpaired t-test, p = 0.4).

We also tested NMDARs and found that although a reduction in the total levels of synaptically localized GluN2A was observed (Figure 5B) (GluN1: WT = 100 ± 10.8%, GluK2(Q) = 106.1 ± 26.7%, unpaired t-test, p = 0.8; GluN2A: WT = 100 ± 17.7%, GluK2(Q) = 44.2 ± 5.2%, unpaired t-test, p = 0.02), no changes were detected in the synaptic expression of GluN2A or the obligatory NMDAR subunit GluN1^63^ in GluK2(Q) mice (Figure 5B) (GluN1: WT = 100 ± 11.2%, GluK2(Q) = 70.6 ± 11.2%, unpaired t-test, p = 0.09; GluN2A: WT = 100 ± 5.5%, GluK2(Q) = 87.7 ± 8.2%, unpaired t-test, p = 0.2).

These data indicate that impairments in GluK2 Q/R editing alter synaptic levels of both KARs and AMPARs, but not NMDARs.

### Impaired LTP at Schaffer collateral-CA1 synapses in GluK2(Q) mice

**Figure 6:**
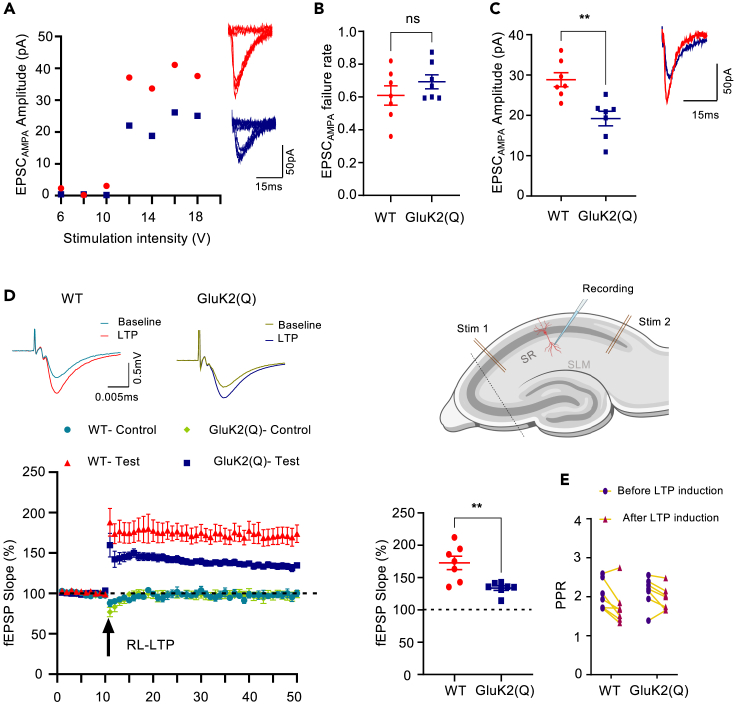
Impaired LTP at Schaffer collateral-CA1 synapses in GluK2(Q) mice (A) Representative data from a minimal stimulation experiment in CA1 showing stimulation intensity threshold for evoking responses from excitation of a single axon fiber (left). Eight superimposed consecutive traces showing synaptic successes and failures for minimal stimulation for WT (red) (top right) and GluK2(Q) (blue) mice (bottom right). (B) Quantification of probability of failures for AMPAR responses out of 150 stimulations in WT and GluK2(Q) mice. N = 6, n = 7 cells for WT and N = 5, n = 7 cells for GluK2(Q) mice; ns p > 0.05; unpaired t-test with Welch’s correction. (C) Quantification of average amplitude of EPSC_AMPA_ (left). Representative trace showing EPSC_AMPA_ in WT and GluK2(Q) mice (right). N6, n = 7 cells for WT and N = 5, n = 7 cells for GluK2(Q) mice; ns p > 0.05, ∗p < 0.05, ∗∗p < 0.01; unpaired t-test with Welch’s correction. (D) Representative traces showing field EPSPs (fEPSP) before and after LTP induction (21–30 min) in WT (top left) and GluK2(Q) mice (middle). Schematics of mouse hippocampal slice, demonstrating CA1, CA3 and positions of recording and stimulating electrodes (top right). Timeline showing fEPSP slope expressed as percentage of baseline subjected to Ripple-Like (RL)-LTP induction (arrow) (bottom left). Normalized fEPSP slope in test pathway 21–30 min after LTP induction in WT and GluK2(Q) mice (bottom right). N = 4 animals, n = 7 cells; ns p > 0.05, ∗∗p < 0.002, ∗∗∗p < 0.0002, ∗∗∗∗p < 0.0001; Un-paired t-test with Welch’s correction. (E) Paired-pulse ratio in WT and GluK2(Q) mice before and after LTP induction. N = 4 animals, n = 7 cells; ns p > 0.05, ∗∗p < 0.002, ∗∗∗p < 0.0002, ∗∗∗∗p < 0.0001; two-way ANOVA with Sidak’s multiple comparison test.

Since we observed a global reduction in the levels of AMPARs in the GluK2(Q) mice, we next characterized CA1 synapses by stimulation of a single presynaptic Schaffer-collateral axon at 1Hz in acutely prepared hippocampal slices.^49^ Postsynaptic responses were recorded from CA1 pyramidal neurons in whole-cell voltage clamp configuration in the presence of picrotoxin (50μM) and D-APV (50μM) to block GABA_A_Rs and NMDARs. As described for MF-CA3 synapses (Figure 1), stimulation intensity of presynaptic axons was increased until a postsynaptic response was observed (Figure 6A). The percentage of trials that failed to evoke a synaptic response (failure rate) was the same for both WT and GluK2(Q) mice (Figure 6B) (WT = 0.60 ± 0.05%, GluK2(Q) = 0.69 ± 0.04%; unpaired t-test, p = 0.2). However, consistent with MF-CA3 synapses and the loss of AMPARs from the synaptosomes, the average AMPAR-EPSC amplitude for successful trials was reduced in GluK2(Q) mice (Figure 6C) (WT = 28.8 ± 1.7pA, GluK2(Q) = 19.2 ± 1.8pA; unpaired t-test, p = 0.002).

These results further confirm the role of GluK2 Q/R editing in maintaining AMPAR-mediated synaptic transmission and indicate that this phenomenon occurs at different hippocampal synapses.

Since GluK2 editing prevents Ca^2+^ entry through the KAR ion channel we wondered if predominant expression of unedited GluK2(Q), which traffic more efficiently and gate Ca^2+^, would reveal EPSC_KAR_ at Schaffer collateral synapses. However, no EPSC_KAR_ were detected following bursts of 3 stimuli at 167Hz given to Schaffer collaterals to drive multiple axons (Figure S2).

We next tested if the reduced synaptic expression of GluA1-and GluA3-containing AMPARs observed in GluK2(Q) mice impacted on the expression of long-term potentiation (LTP). We have previously shown that sharp-wave/ripple like (RL) patterns of activity induce LTP at Schaffer collateral-CA1 synapses that is expressed by increased AMPAR at the synapse, and therefore chose this synapse for these experiments.^24^ Extracellular field potential recordings from acute hippocampal slices revealed that high frequency stimulation that replicates the *in vivo* patterns of hippocampal RL activity^24^^,^^64^ induced robust LTP of AMPAR-mediated EPSPs in WT mice but LTP was significantly reduced in GluK2(Q) mice (Figure 6D) (WT: 172.8 ± 10.4% in test pathway vs. 97.7 ± 3.8% in control pathway; unpaired t-test, p = 0.0002; GluK2(Q): 133.8 ± 3.6% in test pathway vs. 97.2 ± 4.9% in control pathway; unpaired t-test; p = 0.0001; comparison between test pathways, unpaired t-test, p = 0.0087). The paired-pulse ratio remained unchanged after induction of LTP in both WT and GluK2(Q) mice (Figure 6E) (WT = 2.04 ± 0.14 baseline, 1.74 ± 0.17 after LTP, p = 0.325; GluK2(Q) = 2.13 ± 0.14 baseline, 1.95 ± 0.11 after LTP, p = 0.65; two-way ANOVA with Sidak’s multiple comparison test). These data suggest that preventing KAR Q/R editing reduces basal synaptic AMPAR expression and impairs the synaptic recruitment of AMPARs required for LTP.

## Discussion

GluK2 Q/R editing is developmentally^40^^,^^65^ and activity-dependently regulated^35^^,^^36^ to modulate and accommodate distinct synaptic and network diversity. However, how GluK2 editing impacts on KAR function and subsequent downstream AMPAR function has not been explored. To address these outstanding questions we compared WT mice, which contain <15% unedited GluK2(Q), and GluK2 Q/R editing deficient (GluK2(Q)) mice that have >95% unedited GluK2(Q).^44^

In recombinant expression systems KARs containing unedited GluK2(Q) gate Ca^2+^ and have a much greater single channel conductance than those containing edited GluK2(R) (∼150ps compared to <10ps).^43^ We therefore predicted that postsynaptic ionotropic KAR function would be enhanced in GluK2(Q) mice. We found this to be the case, but the increase in EPSC_KA_ we observed (WT = 4.66 ± 0.63pA, GluK2(Q) = 7.31 ± 0.50pA) was markedly less than expected based on the data from heterologous expression systems. However, it should be noted that, to our knowledge there is no available information in the literature on how heteromeric KARs containing GluK2(Q) or GluK2(R) subunits behave when combined with GluK4/GluK5 in *invivo* conditions. Moreover, recombinant KARs in heterologous cell lines generally lack auxiliary subunits and/or interacting proteins (e.g., Netos, KRIP6) that play crucial roles in regulating KAR localization, gating, and function.^1^ Thus, it is likely that cell-line based recombinant KAR systems exhibit different properties to endogenous KARs in primary cultured neurons or cultured *ex vivo* slices.

We also anticipated that the increased conductance and Ca^2+^ permeability of GluK2(Q)-containing KARs should boost presynaptic KAR function, resulting in enhanced short-term facilitation at both 50ms (PPF) and 1s (FF) timescales. This is indeed what we found in the mice, but we also observed an increase in failure rate in the minimal stimulation experiments. These data suggest an additional factor of reduced basal probability of release, or number of release sites, within the large presynaptic mossy fiber boutons, which on its own is predicted to increase presynaptic facilitation. Minimal stimulation at 3Hz is sufficient to engage KAR-mediated FF and would therefore be expected to produce a lower failure rate in GluK2(Q) mice. We observed the opposite effect, suggesting that the reduction in basal probability of release is substantial. Our data cannot distinguish between reduced probability of release and reduced number of release sites so further anatomical investigation would be necessary to address this. Nonetheless, overall, our data indicate that GluK2(Q) mice exhibit enhanced pre- and postsynaptic KAR function.

In stark contrast to their enhanced ionotropic KAR function, GluK2(Q) mice show reduced metabotropic function measured by inhibition of I_sAHP_ at MF-CA3 synapses.^16^^,^^25^^,^^26^^,^^60^ Since I_sAHP_ controls the excitability of CA3 pyramidal neurons and response to synaptic stimulation,^26^ GluK2(Q) is predicted to cause a reduction in the regulation of synaptic integration by KARs. Possible explanations for the diminished metabotropic KAR signaling in GluK2(Q) mice include a reduction in expression of GluK1 and GluK2 KAR subunits and/or Q/R editing state-dependent conformational changes which regulate metabotropic signaling. It remains unclear and controversial which, and how, specific KAR subunits contribute to KAR metabotropic signaling. Indeed, it has been proposed by different groups that GluK1, GluK2, or GluK5 are required for G protein coupling and metabotropic effects.^16^^,^^21^^,^^24^^,^^34^^,^^66^ Thus, although we cannot draw definitive mechanistic conclusions, our data demonstrate that GluK2(Q) mice show reduced metabotropic KAR signaling and, at the same time, enhanced ionotropic function.

Pre- and postsynaptic KARs induce bidirectional plasticity.^6^^,^^8^^,^^24^^,^^38^^,^^67^ Presynaptic KARs at MF-CA3 synapses induce LTD that is sensitive to Ca^2+^ levels suggesting a direct activation of ionotropic KARs or activation of voltage-gated Ca^2+^ channels through metabotropic KAR signaling.^68^ A critical role for postsynaptic KAR signaling is activity-dependent regulation of both KAR and AMPAR surface expression. For example, depending on the extent of activation, KARs can enhance or reduce AMPAR surface expression to evoke LTP or LTD via metabotropic or ionotropic signaling, respectively.^24^^,^^38^ Our data also show that GluK2 editing impacts on NMDAR-induced LTP at Schaffer collateral synapses in CA1, with GluK2(Q) mice exhibiting reduced LTP. GluK2(Q) mice also had reduced AMPAR-EPSCs at mossy fiber and Schaffer collateral synapses and lower GluA1 and GluA3 levels in synaptosomal fractions, suggesting that KARs not only regulate activity-dependent AMPAR trafficking but may also maintain basal synaptic levels of Ca^2+^-permeable AMPARs.

It should be noted that activity-dependent regulation of KAR alters the synaptic expression of GluA1 and GluA2 AMPARs, while GluK2 editing specifically alters the basal synaptic expression of GluA1 and GluA3 containing AMPARs.^24^^,^^38^ Importantly, these changes were specific to AMPARs, since synaptic levels of the NMDAR subunits GluN1 and GluN2A were unchanged, indicating the loss of AMPARs is not due to wholescale changes in synaptic composition in the editing-deficient mice.

These findings demonstrate that KAR activity mediates the ‘tone’ of synaptic AMPARs at MF-CA3 and Schaffer collateral synapses and suggests a wider role where KAR signaling may set the tone for synaptic AMPAR composition more broadly. We speculate that this may be a homeostatic mechanism in which the presence of high-conductance, Ca^2+^-permeable GluK2(Q)-containing KARs causes a compensatory decrease in Ca^2+^-permeable GluA1/GluA3-containing AMPARs to balance synaptic responsiveness.

Unlike recombinant systems or manipulated primary neuronal cultures, the transgenic mice we use have a germline mutation. Thus, the overwhelming majority of GluK2 is unedited throughout development. Therefore, the downregulation of KAR subunit synaptic expression could be a homeostatic mechanism to minimize the excitotoxic effects of Ca^2+^ entry through the Ca^2+^-permeable receptors. While we did not assess how AMPAR subunit expression and activity is altered through development in GluK2(Q) compared to WT mice, we suggest that this may correlate with the observed effects of KAR signaling on expression of AMPARs during the development of synaptic circuits,^50^^,^^69^ and our results indicate that this developmental regulation may extend into adulthood. Furthermore, although we have not investigated this directly, it remains possible that altered KAR signaling and reduction in GluA1 and GluA3 AMPAR subunits could impact neuronal development and network formation.

In healthy adult brain GluK2-containing KARs predominantly comprise edited GluK2(R).^70^ Using mice that almost exclusively express only GluK2(Q) we show that the ionotropic/metabotropic balance of KAR signaling is radically altered by a lack of GluK2 editing. Based on these results, we propose that unedited GluK2(Q)-containing KARs primarily or exclusively function as ion channels with enhanced conductance for both mono and/or di-valent cations, whereas the edited GluK2(R)-containing KARs act as metabotropic receptors to regulate and maintain network activity.

These findings are important because GluK2 Q/R editing is subject to both developmental and activity-dependent control.^36^ Moreover, it has been reported that the proportion of edited GluK2(R) is increased to 85% in patients with a pharmaco-resistant temporal lobe epilepsy (TLE),^70^ raising the possibility that increased inhibition of I_sAHP_, and thereby hyperexcitability, could underpin seizure generation. Conversely, reduction in KAR-mediated inhibition of I_sAHP_ in GluK2(Q) mice might render protection against spontaneous seizures.

Taken together our data indicate that physiologically and pathologically relevant alterations in GluK2 editing may dynamically regulate KAR function, signaling mode, maintenance of functional neuronal networks, and set the threshold for the induction of plasticity. Thus, in conclusion, our results highlight that GluK2 Q/R editing acts as a previously unsuspected molecular switch that regulates the enigmatic dual-mode capability of KARs to operate via either ionotropic or metabotropic signaling, to initiate distinct and diverse downstream pathways.

### Limitations of the study

We are mindful that this study is mainly electrophysiological and therefore does not address the molecular pathways and mechanisms underlying altered KAR signaling in the absence of GluK2 Q/R editing, and the subsequent reduction in synaptic expression of the GluA1 and GluA3 AMPAR subunits. In addition, although we show altered synaptic expression of KAR subunits, further investigations are required to understand how Q/R editing modulates the composition of functional receptors. We further reveal a reduction in GluA1 and GluA3 subunits, likely indicating that KAR-meditated signaling specifically regulates Ca^2+^ permeable AMPARs. However, further investigations will be required to address these questions directly.

We believe the data presented provide compelling evidence that GluK2 Q/R editing significantly regulates KAR signaling and synaptic functions at various synapses in the hippocampus. This work, however, is mainly restricted to young animals (P14-P21), and we have yet to identify how a lack of GluK2 Q/R editing affects synaptic function in adults. Thus, a detailed investigation of the molecular pathways, receptor composition, developmental regulation, and modulation of neuronal excitability by I_SAHP_ provide exciting avenues for future research to understand the role of KAR editing in various neurological and neurodevelopmental disorders, and its potential for future therapeutic intervention.

## STAR★Methods

### Key resources table

**Table:** 

REAGENT or RESOURCE	SOURCE	IDENTIFIER
**Antibodies**		

Rabbit Polyclonal Anti-Glutamate receptor 1	Millipore	RRID: AB_2113602
Mouse Monoclonal Anti-Glutamate Receptor	BD Pharmingen	556341
Rabbit Polyclonal Anti-GluA3	Alomone	RRID: AB_2039883
Rabbit Polyclonal Anti-GluR5	Millipore	RRID: AB_2279352
Rabbit Monoclonal Anti-GluR6/7,clone NL9	Millipore	RRID: AB_1587072
Rabbit Polyclonal Anti-KA2/GRIK5	Millipore	RRID: AB_310100
Rabbit Monoclonal Anti-Neto1	a kind gift from Prof. Susumu Tomita (Yale, USA)	NA
Rabbit Monoclonal Anti-Neto2	Abcam	RRID: AB_10863520
Rabbit Monoclonal Anti-NMDAR1	Abcam	RRID: AB_10862307
Rabbit Monoclonal Anti-NMDAR2A	Abcam	RRID: AB_10975154
Mouse Monoclonal Anti β-actin	Sigma	RRID: AB_47674

**Chemicals, peptides, and recombinant proteins**		

D-AP5	Tocris	0106
GYKI 53655	Hellobio	HB0312
UBP 310	Tocris	2079
Picrotoxin	Sigma-Aldrich	P1675
CGP55845 hydrochloride	Hellobio	HB0960
DCG-IV	Tocris	0975/1
Sucrose	Merck	S038
NaCl	Merck	S3014
KCl	Merck	P5405
NaHCO3	Merck	S5761
NaH2PO4	Merck	S3139
D-Glucose	Merck	G7021
CaCl2	Merck	C5670
MgCl2	Merck	M4880
HEPES	Merck	H4034
EGTA	Merck	324626
CsMeSO4	Merck	C1426
MgATP	Merck	A9187
NaGTP	Merck	G3776
QX314.Cl	Tocris	2313/50
Spermine	Merck	S4264
K-Gluconate	Merck	P1847
Syn-PER™ Synaptic Protein Extraction Reagent	ThermoFisher	87793

**Experimental models: Organisms/strains**		

129Sv ECS	University of Bristol Animal Services	NA

**Software and algorithms**		

CED Signal 5	{Anderson, 2007 #831}	https://www.winltp.com/
GraphPad Prism version 9.3	This paper	https://www.graphpad.com/scientific-software/prism/
LI-COR Biosciences ImageStudio Lite Version 5.2	{Evans, 2019 #47162}.	https://www.licor.com/bio/image-studio-lite/download

**Other**		

Vibratome	Leica	VT 1200s

### Resource availability

#### Lead contact

Further information and requests for resources and reagents should be directed to and will be fulfilled by the Lead Contact, Jeremy Henley (j.m.henley@bristol.ac.uk).

#### Materials availability

This study did not generate new unique reagents.

### Experimental model and study participant details

GluK2 editing-deficient ECS mice and their WT counterparts (129Sv strain) were created at the Salk Institute by mutating the intronic editing complementary sequence (ECS) in the *grik2* gene that directs ADAR2-mediated codon substitution in the GluK2 pre-mRNA.^44^

The mice were housed in groups of 2–4 in standard individually ventilated (IVC) cages in rooms with temperature maintained between 19°C and 23°C and with 12h light and dark cycles. Cages had sawdust, paper nesting and an enriched environment (wooden chews, cardboard tubes etc.). Pups of age P14-P21 were used for the experiments irrespective of sex.

All the animal experiments and procedures were performed in compliance with the UK Animal Scientific Procedures act (1986) and were guided by the Home Office Licensing Team at the University of Bristol. All animal procedures relating to this study were approved by the Animal Welfare and Ethics Review Board at the University of Bristol (approval number UIN/18/004).

All experiments and analysis were performed blinded to mouse genotype.

### Method details

#### Acute hippocampal slice preparation

Cervical dislocation followed by decapitation were performed on P14-21 male and female WT and GluK2(Q) mouse pups. The brain was removed and placed in ice-cold sucrose slicing solution (in mM: Sucrose, 205; KCl, 2.5; NaHCO_3_, 26; NaH_2_PO_4_, 1.25; D-Glucose, 10; CaCl_2_, 0.5; MgCl_2_, 5) saturated with 95% O_2_ and 5% CO_2_. Hippocampi were carefully removed and transverse sections of 400μm thickness for whole-cell recordings and 500 μm for field recordings were obtained using a vibratome. Slices were kept for recovery in a slice holder containing artificial cerebrospinal fluid (aCSF; in mM: NaCl, 124; KCl, 3; NaHCO_3_, 24; NaH_2_PO_4_, 1.25; D-Glucose, 10; MgSO_4_, 4; CaCl_2_, 4) for whole-cell recordings and (aCSF; in mM: NaCl, 124; KCl, 3; NaHCO_3_, 24; NaH_2_PO_4_, 1.25; D-Glucose, 10; MgSO_4_, 2; CaCl_2_, 2) for field recordings saturated with 95% O_2_ and 5% CO_2_ at 37°C for 20 min and later transferred to room temperature for at least 30 min before performing experiments.

#### Electrophysiology recordings

##### Whole cell recordings

Hippocampal slices were placed in a submerged holding chamber continuously perfused with oxygenated aCSF at 36.5°C at a flow rate of 3mL per minute. Hippocampal CA3 pyramidal cells were visually identified using DIC optics and patch-clamped in whole-cell configuration using a pulled Harvard borosilicate glass capillary of resistance 5-7M MΩ filled with either caesium-based whole-cell solution (in mM: NaCl, 8; CsMeSO_4_, 130; HEPES, 10; EGTA, 0.5; MgATP, 4; NaGTP, 0.3; QX314.Cl, 5; Spermine, 0.1) or K-Gluconate based (in mM: NaCl, 8; KGluconate, 135; HEPES, 10; EGTA, 0.2; MgATP, 2; NaGTP, 0.3) for I_sAHP_ experiments.

The cells were held in voltage clamp mode and evoked EPSCs were obtained by stimulating the mossy fiber pathway with a bipolar stimulating electrode placed in the dentate gyrus hilus layer (or glass monopolar electrode for minimal stimulation experiments). Picrotoxin (50μM) was included in the aCSF to inhibit GABA_A_ receptors (except for CA1 field recordings). Cells with series resistance above 30 MΩ or where series resistance changed by >20% were excluded from analysis. To confirm the purity of mossy fiber inputs, the group-II mGluR agonist DCG-IV (2μM) was bath applied for 5–10 min at the end of experiments with mossy fiber stimulation.^26^ Recordings were only included in analysis if DCG-IV reduced EPSCs by >70%.

##### Minimal stimulation

###### CA3 pyramidal cells

Cells were voltage clamped at −60mV and MF-EPSCs were evoked by moving a mono-polar stimulating electrode filled with caesium-based whole-cell solution (in mM: NaCl, 8; CsMeSO_4_, 130; HEPES, 10; EGTA, 0.5; MgATP, 4; NaGTP, 0.3; QX314.Cl, 5; Spermine, 0.1) around the inner border of dentate gyrus granule cells until a response was observed. Stimulation intensity was adjusted just above the threshold for activation of a synaptic response. Consecutive traces were recorded at a frequency of 3Hz. No prominent polysynaptic activation was observed using this low intensity stimulation. AMPAR-EPSCs were measured for 5–10 min in the presence of D-APV (50μM) for a minimum of 150 trials. Subsequently, GYKI53655 (40μM) was applied to block AMPAR responses and KAR-EPSCs were measured after 15 min of GYKI53655 application and for 5–10 min and a minimum of 150 trials.

###### CA1 pyramidal cells

Cells were voltage clamped at −60mV and Schaffer collaterals were evoked by moving a mono-polar stimulating electrode filled with caesium-based whole-cell solution (in mM: NaCl, 8; CsMeSO_4_, 130; HEPES, 10; EGTA, 0.5; MgATP, 4; NaGTP, 0.3; QX314.Cl, 5; Spermine, 0.1) around the stratum radiatum until a response was observed. Stimulation intensity was adjusted just above the threshold for activation of a synaptic response. Consecutive traces were recorded at a frequency of 1Hz. No prominent polysynaptic activation was observed using this low intensity stimulation. AMPAR-EPSCs were measured for 5–10 min in the presence of D-APV (50μM) for a minimum of 150 trials.

##### KAR/AMPAR ratio

CA3 pyramidal neurons were voltage clamped at −60mV in the presence of D-APV (50μM). Mossy fibers were stimulated with a burst of 3 stimuli at 167Hz every 20s to evoke AMPAR/KAR-EPSCs. Stable AMPAR-EPSCs were recorded for 20 min and then KAR-EPSCs were recorded for 20 min in the presence of the AMPAR antagonist GYKI53655 (40μM).

##### Paired-pulse and frequency facilitation

CA3 neurons in acute hippocampal slices were voltage clamped at −70mV in the presence of picrotoxin (50μM). To measure PPF, EPSCs were evoked by pairs of stimuli to mossy fibers at an inter-stimulus interval of 50ms, every 20s. For FF experiments, single stimuli were given at 0.05Hz for 10 min before stimulation frequency was increased to 1Hz for 1 min. After this, stimulation frequency was returned to 0.05Hz. DCG-IV (2μM) was then applied for at least 5 min to assess purity of MF input.

##### Slow afterhyperpolarizations (I_sAHP_)

For I_sAHP_ recordings, CA3 pyramidal neurons were voltage clamped at −50mV in the presence of picrotoxin (50μM), D-APV (50μM), CGP55845 (1μM) (Hello Bio: HB0960) and GYKI53655 (40μM). Glass electrodes were filled with whole-cell solution (in mM: NaCl, 8; KGluconate, 135; HEPES, 10; EGTA, 0.2; MgATP, 2; NaGTP, 0.3). I_sAHP_ were induced every 20s by applying a depolarising voltage step to 0mV for 200ms and I_sAHP_ amplitude was measured 300ms after returning the membrane potential to −50mV to avoid measurement of medium afterhyperpolarization (I_mAHP_). Synaptic activation of KARs was induced by bursts of 10 stimuli at 25Hz to the mossy fibers 500ms prior to the induction of I_sAHP_.

##### Field potential recordings

Extracellular field potentials (fEPSPs) were recorded from stratum radiatum in CA1 using a 3–5 MΩ glass pipette filled with aCSF. Two stimulation electrodes (bipolar) were positioned on opposite sides of the recording electrodes equidistant from the pyramidal layer to evoke two independent inputs (Stim1 and Stim2). LTP induction protocol was delivered only to Stim1 and was alternately positioned closer to the CA3 region or to subiculum in different recordings. Paired stimuli (50ms inter-stimulus interval) were given every 10s to each pathway, alternating between the control and test pathway (Stim1 and Stim2). LTP consisted of 20 bursts of 20 stimuli at 200Hz given every 5s. The recordings were performed in the absence of picrotoxin.

#### Synaptosomal preparations and Western blotting

Synaptosomes were prepared from cerebral hemisphere of P14-21 pups using Syn-PER Reagent. After cervical dislocation followed by decapitation of the pups, the brain was removed and cut into two-halves (along the cerebral hemispheres). The cerebellum was discarded. Each cerebral hemisphere from the pup was weighed and transferred to a glass homogenizer and the required amount of Syn-PER reagent was added to the tissue (10mL of reagent per gram of tissue). The tissue was homogenized on ice with slow stokes (∼10 strokes). The homogenate was transferred to a fresh centrifuge tube and centrifuged at 1200 x g for 10 min at 4°C. The supernatant was transferred to a fresh tube and the pellet was discarded. The supernatant was centrifuged again at 15,000 x g for 20 min at 4°C. The supernatant was discarded, and the pellet was resuspended in 1-2mL of Syn-PER reagent. To the synaptosomal fraction, Triton X-100 and SDS were added to a final concentration of 1% and 0.1%, respectively, and left at 4°C on a rotating wheel for 1h to lyse synaptosomes and solubilize membrane proteins. The samples were then centrifuged at 16,000 x g for 20 min to remove insoluble material. Protein quantification was performed on the samples and the final samples for Western blotting were prepared by adding 2X sample buffer and boiling at 95°C for 10 min.

The samples prepared were separated based on molecular weight using Sodium dodecyl sulphate-poly acrylamide gel electrophoresis (SDS-PAGE). The gel comprised a 10% acrylamide resolving gel (375mM Tris-HCL pH 8.8, 10% acrylamide, 0.1% SDS, 0.1% APS and 0.01% TEMED) and 5% stacking gel (125mM Tris-HCL pH 6.8, 5% acrylamide, 0.1% SDS, 0.1% APS, 0.01% TEMED).The gels were transferred to PVDF membrane and blocked in 5% skimmed milk in PBS-T (0.137M NaCl, 2.7mM KCl, 10mM Na_2_HPO_4_, 2mM K_2_HPO_4_, pH to 7.4 with HCl, 0.001% Tween 20) for 1 h at RT and blotted overnight at 4°C in the same blocking solution with the primary antibody.

### Quantification and statistical analysis

#### Electrophysiology recordings

##### Data acquisition

Data were digitized at 10kHz, and low-pass filtered at 2kHz using CED Micro 1401-4 A-D acquisition unit and Axon patch 200B amplifier (Molecular devices). All recordings were obtained using CED Signal 5 software.

##### Data analysis

CED Signal acquisition software was used to analyze the recorded data. Mean responses were obtained every minute by averaging consecutive traces. EPSC amplitudes were measured from the averaged traces and normalized to the mean EPSC amplitude of baseline.

###### Whole cell recordings

CED Signal acquisition software was used to analyze the recorded data. Mean responses were obtained every minute by averaging consecutive traces. EPSC amplitudes were measured from the averaged traces and normalized to the mean EPSC amplitude of baseline.

####### τ_decay_

**τ**_decay_ for AMPAR and KAR-EPSCs were calculated by single exponential curve fitting feature in CED signal software. The equation used is f(x) = a_0_ e ^−x/a^_1_ + a_2_. Wherein f(x) is regarded as the current as a function of time, a_0_ is the amplitude at time 0 and a1 is the time constant (**τ**) and a_2_ is the steady state current.

###### KAR/AMPAR ratio

Amplitude of AMPAR-KAR peaks and KAR peaks were measured individually by averaging over a period of 5 min before and after GYKI53655 application and the ratio of KAR-EPSC to AMPAR-EPSC were calculated.

###### Paired-pulse and frequency facilitation

. Paired-pulse ratios were obtained by averaging amplitudes of P1 peak to P2 peak.

FF ratios were obtained by averaging the last 20 frames of P1 amplitude at 0.05Hz with the middle 40 frames at 1Hz stimulation.

####### Percentage of DCGIV block

%DCGIV block = (Average baseline P1 peak- Average P1 peak with DCGIV)/Average P1 peak ∗100)

###### Field potential recordings

fEPSP slopes were measured using CED signal software. The slopes are displayed as a percentage of 10 min baseline. For LTP quantification the values were obtained 21–30 min after LTP induction.

###### Western blotting

For each experiment, the signal for each condition was divided by the signal from the loading control for that experiment (β-actin). This analysis was performed for each replicate experiment, and for presentation purposes, the mean of the WT is set to 100%.

#### Statistical analysis

Data are represented as mean ± SEM. ‘N’ - Number of animals used, ‘n’ - number of cells. ANOVA, or paired or un-paired Student’s *t* test were used for statistical analysis and stated in the figure legends with respective p values. All statistical tests were performed using GraphPad Prism version 9.3.

## Data Availability

• **Data**: All data reported in this paper will be shared by the lead contact upon request. • **Code**: This paper does not report original code. • **Experimental model and study participant details**: The following animals were generated in salk institute, obtained from Prof Bryce Vissel (Center for Neuroscience and Regenerative Medicine, St Vincent’s Hospital, Sydney, Australia). The animals are currently available at the animal services facility at the University of Bristol. • **Any additional Information**: Any additional information required to reanalyze the data reported in this work paper is available from the lead contact upon request.
