# Supplementary material for: GluK2 Q/R editing regulates kainate receptor signaling and long-term potentiation of AMPA receptors
Source: iScience. 2023 Aug 25;26(10):107708. doi: 10.1016/j.isci.2023.107708 (PMC10504484; doi:10.1016/j.isci.2023.107708)
Supplement: Document S1. Figures S1 and S2 [file mmc1.pdf]

iScience, Volume *26*

Supplemental information

GluK2 Q/R editing regulates kainate receptor signaling and long-term potentiation of AMPA receptors

Jithin D. Nair, Kevin A. Wilkinson, Busra P. Yucel, Christophe Mulle, Bryce Vissel, Jack Mellor, and Jeremy M. Henley

Figure S1

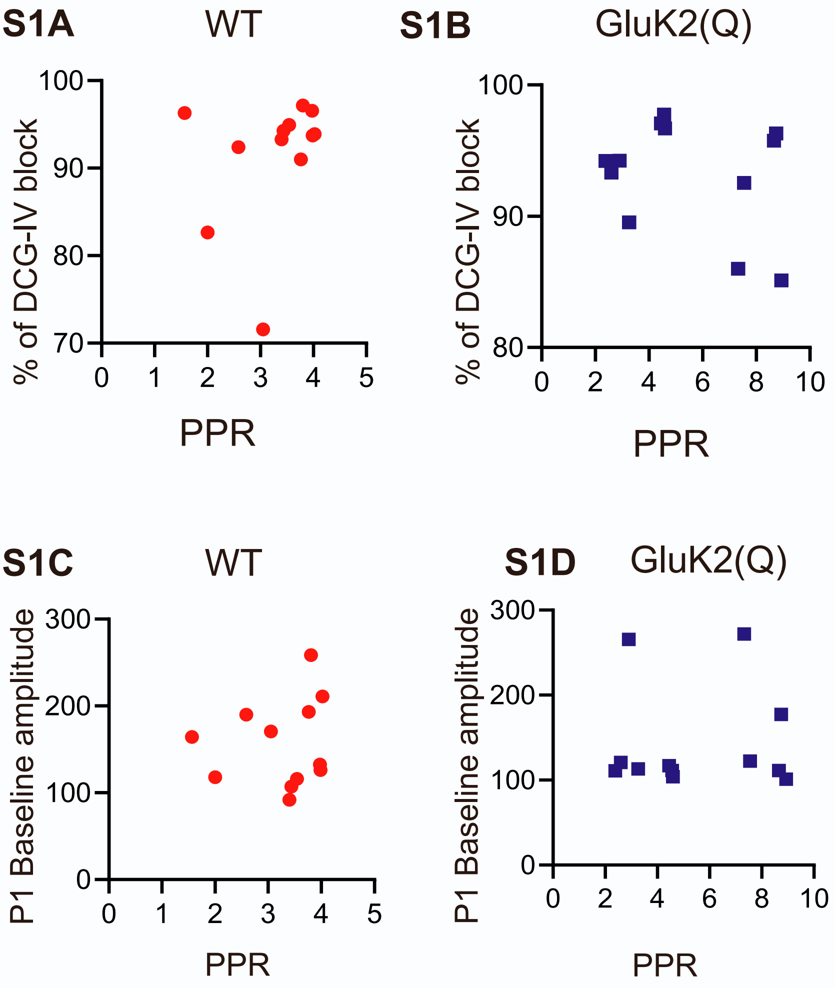

Supplementary Figure S1: Correlation (Related to Figure 2)

Pearson's correlation between PPR and percentage of DCG-IV block in WT **(S1A) and** GluK2(Q) mice **(S1B)**. PPR and P1 baseline amplitudes of WT **(S1C)** and GluK2(Q) mice **(S1D).** WT, N=7, n=13 cells; Tg, N=8, n=15 cells.

Figure S2

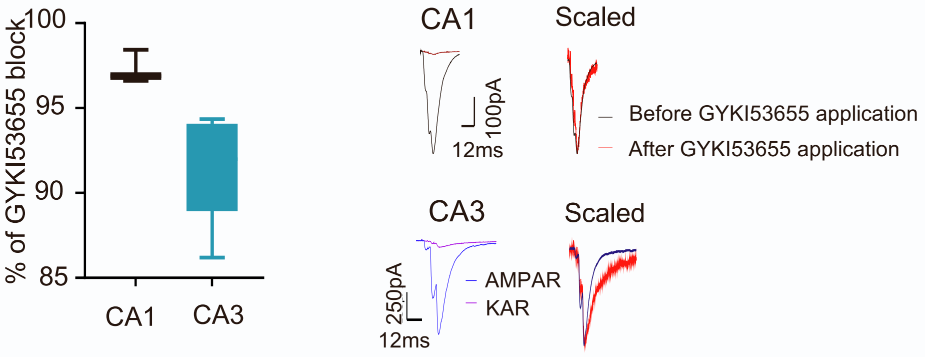

Supplementary Figure S2: No KAR currents in CA1 synapses of GluK2(Q) mice (Related to Figure 6)

Quantification of percentage of GYKI53655 block in CA1 and CA3 synapses in GluK2(Q) mice **(left)**. Representative traces showing postsynaptic EPSCs elicited by bursts of stimuli in CA1 **(top middle)** and CA3 **(bottom middle)** of GluK2(Q) mice before and after 10 mins of GYKI5655 (40μM) application to block AMPA receptors. A larger percentage of the response remains after GYKI53655 in CA3. Scaled response before and after GYKI53655 application in CA1 **(top right)** and CA3 **(bottom right)**. The remaining CA1 currents show identical decay kinetics indicating a residual AMPAR response, while CA3 currents post-GYKI53655 application show a slow decay kinetics consistent with KAR responses.
